# The epidemiology of canine ear diseases in Northwest China: Analysis of data on 221 dogs from 2012 to 2016

**DOI:** 10.14202/vetworld.2023.2382-2388

**Published:** 2023-11-27

**Authors:** J-P Li, L-Y Li, F-L T, D-Z Lu

**Affiliations:** 1 Department of Clinical Veterinary Medicine, College of Veterinary Medicine, Northwest A&F University, Yangling, Shaanxi, 712100, P.R. China; 2 Xi’an Teaching Hospital, Northwest A&F University, Xi’an, Shaanxi, 71007, P.R. China

**Keywords:** dog, epidemiology, otitis externa, Northwest of China

## Abstract

**Background and Aim:**

Ear disease is relatively important in veterinary medicine as it significantly affects the quality of life of pets. Two hundred and twenty-one cases of canine ear diseases were collected and collated at the Xi’an Teaching Hospital of Northwest A&F University from 2012 to 2016. An epidemiological analysis was conducted to evaluate the prevalence and causes of various ear diseases in various breeds of dogs in Xi’an.

**Materials and Methods:**

Data were collected and statistically analyzed by reviewing previous cases and obtaining medical history data and laboratory results. This study included the following experiments: systematic examination of the animals’ skin, auricular skin scraping test, ear canal endoscopy, and examination of ear canal secretion.

**Results:**

The top three dog breeds most commonly afflicted with ear diseases in Xi’an were Toy poodles, Cocker Spaniels, and Golden Retrievers, accounting for 18.5%, 10.4%, and 9.5% of the total cases, respectively. The prevalence was the highest in August and September, with male dogs having a higher prevalence rate than female dogs. Common ear diseases were categorized as otitis externa, otitis media, otitis interna, or ear hematoma.

**Conclusion:**

This study determined the prevalence of external otitis, ear hematoma, otitis media, and inner otitis in dogs in the Xi’an area. These results can help expand the current understanding of the development and epidemiology of canine ear diseases and provide a reference for clinical diagnosis, treatment, and prevention.

## Introduction

As the quality of life in Xi’an continues to improve, an increasing number of people have taken the option of keeping pets. However, ear diseases are common afflictions in pets, especially in dogs. The characteristics of ear disease include pruritus, a long treatment period, and ease of recurrence. If treatment is not timely, secondary otitis media and otitis interna may occur, ultimately affecting the dog’s hearing. In severe cases, neurological symptoms may develop and negatively affect the lives and health of dogs. Furthermore, a delay in diagnosis, resulting in a delay in appropriate treatment, increases the morbidity and mortality of otitis media and internal otitis [1].

This study aimed to analyze the prevalence and causes of ear diseases in various dog breeds in Xi’an. We collected and analyzed 221 cases at Xi’an Teaching Hospital of Northwest A&F University from 2012 to 2016. We classified them into four categories (otitis externa, otitis media, otitis interna, and ear hematoma) for the epidemiological analysis.

This study is the first to develop the prevalence for analyzing canine ear diseases in Xi’an. These findings can help expand our current understanding of the development and epidemiology of canine ear diseases and provide a reference for clinical diagnosis, treatment, and prevention.

## Materials and Methods

### Ethical approval

This study was approved by the Research Ethics Committee, Northwest A&F University (Approval no. XN2022-1008).

### Study period and location

Data on canine ear disease at Xi’an Teaching Hospital of Northwest A&F University from 2012 to 2016 were collected and statistically analyzed by reviewing previous cases and obtaining medical history data and laboratory results.

### Examination of ear diseases

The skin of the animals was examined systematically. The ears were first examined using an otoscope, and physical features were recorded: each ear was then sampled with an individual swab to determine whether the infection was bilateral or unilateral. The dry swab was inserted deep enough to reach the junction between the horizontal and vertical ear canals and rotated several times. The swab was gently rolled on a clean slide, the tip was completely rotated, and the slide was air-dried, fixed with methanol, and stained with Wright’s stain, The sample was examined using a composite microscope [Olympus, (Shenzhen) Industrial Co., LTD, Shenzhen, China] at low magnification (40×). The ears were carefully observed for depilation, callus, dandruff, erythema, and thickening to determine whether the ear canal was moist and whether it had secretions and an odor.

### Auricular skin scraping test

First, the coat was cut at the junction of the diseased skin and healthy skin, and skin scrapings were collected by gently scraping along the direction of the hair with a blunt blade until there was slight bleeding. Skin scrapings were placed on a slide, and Rayleigh’s dye was added to one part of the slide to determine the presence of bacteria under microscopic examination. Two drops of 10% KOH were added to another part of the slide, and the section was covered with a coverslip. The liquid on the slide was heated slightly until transparent and observed by microscopy for the presence of insects.

### Examination of ear canal secretion

Sterile cotton swabs were used to collect ear canal secretions, which were then smeared onto slides. The secretions were observed under a microscope after air drying, methanol fixing, and staining by Wright’s stain. An ear endoscope was used to assess the condition of the ear canal. The probe was inserted to check for any foreign bodies, determine the integrity of the middle ear structure, or biopsy the ear canal tumor. We prepared a wet tablet sample from secretions collected from the ear canal to determine parasitic infection. The presence of ectoparasites was confirmed using a skin smear of the ear canal. The sample was examined using a composite microscope [Olympus, (Shenzhen) Industrial Co.] at low magnification (10×). Counting the number of parasites involved scanning the entire wet sheet from top to bottom and from left to right, according to parasitological identification keys [2].

### Diagnostic criteria for ear diseases [3]

Diagnostic criteria for otitis externa: Otitis externa was diagnosed when the skin of the ear canal was moist, inflamed, swollen, and itchy.

Diagnostic criteria for otitis media: Otitis media was diagnosed based on elevated body temperature, perforation of the eardrum, and purulent discharge.

Diagnostic criteria for inner otitis: Dogs with deafness, imbalance, turning, and head and neck tilt were diagnosed with inner otitis.

Diagnostic criteria for othematoma: Head shaking due to otitis externa and hematoma formation within the cartilage.

## Results

**Figure-1:**
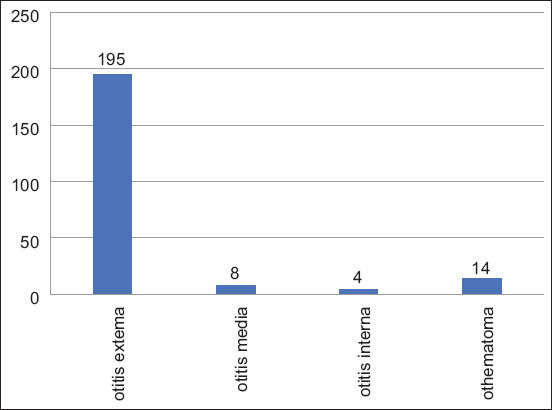
The distribution of canine ear diseases from 2012 to 2016 in Xi’an.

A statistical survey of outpatient veterinary records from Xi’an Teaching Hospital of Northwest A&F University was conducted from January 2012 to December 2016. The total number of dog outpatient cases was 20,404, of which 221 (1.0%) were ear diseases (Table-1). Otitis externa had the highest prevalence at 84.6% (195/221) followed by ear hematoma at 6.3% (8/221). The prevalence of otitis media and otitis interna was 3.6% (4/221) and 1.8% (14/221), respectively (Figure-1).

**Table-1:** The prevalence of canine ear diseases from 2012 to 2016.

Year	2012	2013	2014	2015	2016
Total cases	3224	4417	4196	4563	4004
Number of dogs ear diseases	38	34	48	51	50

**Figure-2:**
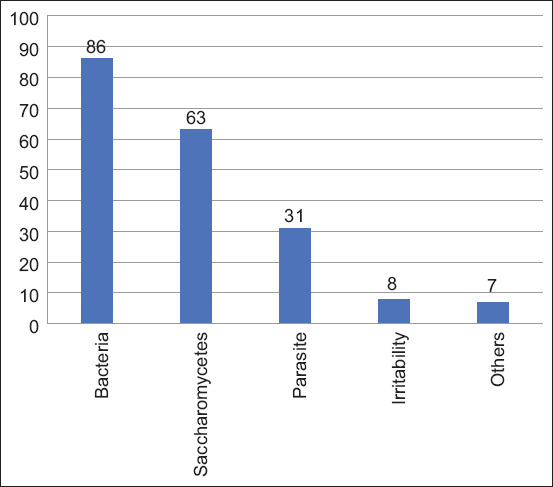
The distribution of causes dog otitis externa from 2012 to 2016 in Xi’an.

Among 195 cases of canine otitis externa, the prevalence of bacterial otitis externa was caused by cocci was the highest, accounting for 86 cases (44.1%); 63 cases were caused by fungi and/or *Malassezia* (32.3%); 31 (15.9%) cases of parasitic otitis externa were caused by mites; and 8 (4.1%) cases of otitis externa caused by foreign bodies or immunoglobulin E (+). Other causes included auricular hyperplasia and ear tumors, which were observed in 7 (3.5%) cases (Figure-2).

**Figure-3:**
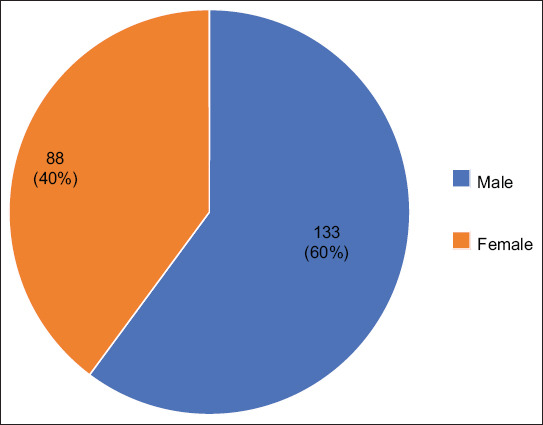
The distribution of canine ear diseases by sex.

The prevalence of canine ear diseases was investigated in three age groups. The prevalence of ear diseases was highest in elderly dogs (>7 years old), accounting for 39.8% of cases, followed by adult dogs (1–7 years old) at 35.7%, and young dogs (<1 year old) at 24.4% (Table-2). Statistical analysis of the three age stages revealed that ear diseases in young dogs were mainly otitis externa, whereas, in adult and elderly dogs, otitis media, otitis interna, ear skin hyperplasia, and ear tumors were more common (Table-3). As shown in Figure-3, the number of male dogs was 133 (60.1%), whereas that of female dogs was 88 (39.8%).

**Table-2:** The distribution of dogs at the three age stages.

Age stages	Number of cases	prevalence (%)
<1 year old	54	24.43
1–7 years old	79	35.75
>7 years old	88	39.82

**Table-3:** The prevalence of common ear diseases in dogs at the three age stages.

Ear disease	Age stages		

	<1 year old	1-7 years old	>7 years old
Otitis externa	52	69	74
Otitis media	0	3	5
Otitis interna	0	2	2
Othematoma	2	5	7

In Xi’an, multiple cases of canine ear diseases were recorded from June to October, with the highest prevalences in August and September (36 and 23 cases, respectively), accounting for 16.2% and 10.4% of ear diseases studied, respectively (Figure-1).

The top three breeds most commonly afflicted with ear disease in Xi’an were Toy poodles, Cocker Spaniels, and Golden Retrievers, accounting for 18.5%, 10.4%, and 9.5% of cases, respectively (Table-4).

**Table-4:** The prevalence of canine ear diseases in different breeds.

Breed	Number	Prevalence	Breed	Number	prevalence
Toy poodle	41	18.55	Bulldog	6	2.71
Cocker spaniel	23	10.41	Huskie	6	2.71
Golden retriver	21	9.5	German Shepherd Dog	6	2.71
Pekingese	13	5.88	Chow	3	1.36
Schnauzer	13	5.88	Sharpei	3	1.36
Mix dog	12	5.43	Rottweiler	2	0.9
Pomeranian	11	4.98	Chihuahua	2	0.9
Labrador retriver	10	4.52	Border Collie	2	0.9
Shiba inu	9	4.07	Yorkshire terrier	2	0.9
Akita	8	3.62	Bichon Frise	2	0.9
Poodle	8	3.62	Doberman Pinscher	2	0.9
Samoyed	7	3.17	Corgi	2	0.9
Shih tzu	6	2.71	Shelties	1	0.5

## Discussion

From 2012 to 2016, the total number of outpatient canine diseases treated at Xi’an Teaching Hospital was 20,404, with an average of 4,080 cases/year. However, the number of canine ear disease cases (221) was lower than that of other diseases, accounting for only 1.0%. Endoscopy is one of the most commonly used detection methods in clinical practice, and it plays a crucial role in determining and treating diseases including ear canal examination [4].

The canine ear disease with the highest prevalence was otitis externa. The external auditory canal is a structure that is directly in contact with the external environment; therefore, it is naturally the most susceptible to injury. The external auditory canal is divided into the vertical and horizontal ear canals. This special structure is conducive to the growth and reproduction of pathogenic microorganisms, resulting in their vulnerability to infection [5]. Ear hematoma mostly develops from otitis externa. When a dog’s ears feel itchy, excessive scratching can lead to an ear hematoma. Increasing age, body weight, and breeds with a V-shaped drop and semi-erect ear carriage increase the odds of aural hematoma. For example, Saint Bernard and French Bulldog are more likely to cause ear hematoma [6]. Similarly, the temperament of some dog breeds, such as the German Shepherd, which are prone to fight and bite, may have a higher prevalence of ear hematoma [7]. Otitis media and interna develop secondarily to otitis externa. In general, only otitis externa is considered serious, and recurrence affects the middle and inner ear structure. In rare cases, the tympanic membrane ruptures, allowing microbes to enter the middle and inner ear.

The etiology of otitis externa cases is generally multifactorial and has been classified according to the primary, secondary, predisposing, and perpetuating (PSPP) system: PSPP factors [8]. Epithelial homeostasis and tissue health are maintained by skin commensals. Bacterial overproliferation can trigger skin inflammation and diseases [9]. As shown in Figure-2, the most common cause of otitis externa was bacterial infection, whereas the most common bacterial genera found in the ear canal were *Staphylococcus*, *Pseudomonas*, and *Proteus*. In this study, the microorganisms in the ear canals of healthy and affected dogs were inoculated into the culture medium for microbial culture identification. Only a few colonies were observed in the cultures of healthy dogs. Conversely, 383 bacterial strains were identified in the cultures of affected dogs. The most commonly cultured strains were *Staphylococcus* (199 strains, 51.9%), *Corynebacterium* (73 strains, 19.0%), and *Streptococcus* (53 strains, 9.1%). Bacteria are ubiquitous in the environment, and under normal conditions, there is a balance in the types of bacteria exist in the ear canal. Changes in the environment in the ear canal can create conditions conducive to bacterial reproduction [10].

The diversity of cutaneous microbiota in dogs appears to decrease in diseased states [11]. The most common cause of a fungal canine ear infection was *Malassezia*. Other fungi also exist in the ear canal, such as *Candida* and *Trichophyton* [12]. *Malassezia* is found in the skin and ear canals of humans and animals and can cause skin diseases and otitis externa [13]. *Malassezia* otitis can present as overgrowth or include inflammatory cells in concurrent exudative bacterial otitis. Excessive growth of Malassezia can lead to inflammatory cell infiltration around the dermal blood vessels leading to inflammation [14]. When inflammation occurs in the ear canal, the free fatty acids on the surface of the ear canal decrease, and the triglyceride concentration increases, creating conducive conditions for *Malassezia* reproduction [15]. Statistically, many canine ear diseases are not single bacterial or fungal infections but are mixed infections. Therefore, multiple pharmacological treatments must be considered.

The third cause of canine ear disease was external parasites, and the main causative agent was *Otodectes cynotis*. Its developmental cycle includes four stages: egg, larva, nymph, and adult. Dogs show clinical symptoms 2–3 weeks after infection [8]. The mite lives on the surface of the ear canal, but does not enter the inner skin layer. However, mouthparts are used to pierce the skin and feed. During feeding, it secretes toxic substances, which causes chemical irritation to the epidermis. Dogs with this infection often present with itchy ears. Because *O. cynotis* has a hard outer layer and is highly resistant to the environment, they’re more likely to survive at lower temperatures and higher humidity, it often survives after leaving the host for several months; therefore, the treatment cycle is relatively long, and the host may be prone to recurrence.

Table-2 shows that the prevalence of ear disease was higher in adult and elderly dogs than in younger dogs. Nevertheless, the prevalence of otitis externa was high at each age stage: 26.6% in young dogs, 35.3% in adult dogs, and 37.9% in elderly dogs. In young dogs, the development of organs is not yet fully mature, and the barrier function and immunity of the skin are still relatively weak. When dogs are brought outdoors, they may be exposed to many parasites and causative agents, such as ear mites, which increase the risk of allergies. In elderly dogs, sebum secretion is primarily influenced by hormones. Older dogs have lower metabolic function and fewer hormones, which means that fewer nutrients can be availed by parasites and opportunists, creating an inhospitable environment for microbial proliferation [16]. Therefore, the prevalence of ear diseases in elderly dogs should be low. However, due to the gradual decline in organ function in elderly dogs, the immunity of the skin is reduced, facilitating the invasion of pathogenic microorganisms [17]. Adult dogs tend to be past the development stage and exhibit high hormone secretion, with the sebaceous glands in the ear canal becoming hypofunctional, creating a suitable environment for the reproduction of microorganisms. If epithelial keratinization in the ear canal is poor, cellular debris cannot be naturally eliminated in time, resulting in the accumulation of otoliths in the ear canal, which can also cause otitis externa.

Among the examined dogs, 133 were male, accounting for 60.1% of the total cases. In the literature, no direct correlation was established between the prevalence of canine ear disease and sex [18]. In the present study, the male-to-female ratio was 1:1.5, and this difference was significant. This difference may be related to hormone secretion. In dogs, secretion of sebaceous glands is affected by hormones. Androgens may lead to hyperplasia and hypertrophy of the sebaceous glands. The exuberant sebum provides a highly nutritious environment for microorganisms. Conversely, estrogen causes degeneration and decline in sebaceous glands; thus, female dogs may have a lower risk of ear disease than male dogs. However, some endocrine diseases may cause abnormal secretion of sebaceous glands, leading to ear disease. Furthermore, the nutritional level of dogs improves with the development of people/s living standards. Excessive meat intake can lead to abnormal exuberant secretion of sebaceous glands, causing clinical symptoms, such as pruritus and swelling [19].

**Figure-4:**
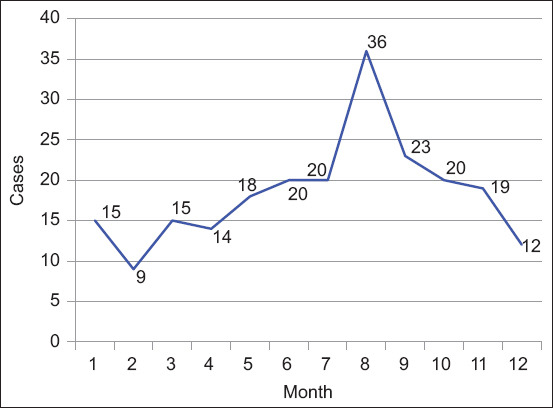
The distribution of canine ear diseases by month.

As shown in Figure-4, the highest prevalence of ear diseases in Xi’an occurred from June to October, accounting for 56.4% of the total cases. August and September were the highest-risk periods, with 36 and 23 cases, respectively, accounting for 16.2% and 10.4% of ear diseases, respectively. Thus, the epidemic seasons of canine ear diseases in Xi’an are mainly summer and autumn. Xi’an is the largest city in northwest China and experiences four distinctive seasons, including hot and rainy summer and cool and rainy autumn. The proportion of precipitation in the summer and autumn accounts for more than 70.0% of the annual precipitation. Many studies have shown that seasonal changes may lead to the development of ear disease. The results of our analysis suggest a relationship between prevalence of canine ear disease and season [20].

Xi’an experiences high rainfall in summer and autumn, with a long rainy period and high air humidity [21]. The structure of the ear canal is relatively closed and a moist environment is beneficial for the growth of microorganisms. In addition, Xi’an also has higher temperatures in summer and autumn. Since dogs have no sweat glands, they cannot eliminate heat through sweat only by panting. The conditions under such circumstances prolong the dilation of capillaries on the body surface of dogs, which increases the chance of parasitic invasion [22]. In summer and autumn, dogs spend more time outdoors and have more opportunities to be exposed to pathogenic microorganisms. Parasites, such as ear mites and fleas, are also more active in summer and autumn [23]. In addition, Xi’an experiences high temperatures in summer and autumn, and dog owners may feel the need to increase the number of baths, which can weaken and even break the dermal barrier, resulting in reduced skin protection and a significant increase in the risk of illness [24]. In general, the degree of hair growth in the external auditory canal may affect the ear canal temperature, which increases the internal temperature and the likelihood of microorganism reproduction. One study tested the effect of ear hair growth on ear temperature in 650 dogs with no clinical manifestations of otitis externa. The study found that the degree of hair growth in the ear canal did not increase but instead decreased the temperature in the ear canal. This is inconsistent with our previous assumptions, causing inconsistencies in the impact of ear hair growth on ear canal temperature and prevalence of ear diseases [25]. Nevertheless, our results indicate that increased skin temperature leads to changes in skin structure and function. However, dermal inflammation in the ear canal was more likely influenced by intrinsic and extrinsic factors rather than by temperature alone [26].

In our study, the prevalence of ear disease in Toy poodles was high at 18.5%. Toy poodles are considered cute and popular among young people in northwest China, which has led to a yearly increase in the number of breeding programs. Thus, the number of ear disease cases has increased accordingly. The Toy poodle breed has more hair in the ear canal, which prevents air circulation and maintains the ear canal in a humid environment. This is conducive to microbial reproduction and otitis externa. Some researchers have pointed out that dogs with drooping auricles are more likely to develop ear diseases if they do not consider hair density in the ear canal as a pathogenic factor [27]. The prevalence of ear disease in cocker spaniels and Golden Retrievers was 10.4% and 9.5%, respectively. The hair in the ear canal is denser than in other breeds, and the two breeds also have large and drooping auricles. Therefore, compared with dogs with vertical auricles, drooping auricles prevents air circulation and heat dissipation in the ear, resulting in a moist environment in the ear canal conducive for the reproduction of microorganisms. The cerumen glands in the ear canal are also abundant. Under bacterial or parasitic stress, the cerumen glands produce a large amount of substances, and the exuberant hair causes these secretions to remain mostly in the ear canal, providing abundant nutrients for microorganisms to grow and reproduce.

However, some breeds with drooping auricles, such as beagle dogs, have a low prevalence of ear diseases, whereas some species with vertical auricles, such as huskies, have a higher prevalence. Our analysis indicates that drooping auricles do not directly cause otitis externa; however, they may affect the efficacy of treatment. Nevertheless, differences in ear anatomy can affect susceptibility to ear diseases. In some breeds, such as Sharkskin and Bulldog, the ear canal structure is congenitally long and narrow and is angled from the horizontal plane, which affects secretion discharge and increases susceptibility to ear diseases. Some conditions, such as ear canal edema, mass, and ear hyperplasia, are acquired. These conditions may put pressure on the ear canal, resulting in stenosis and ear diseases.

The results of our survey differed across regions. This may be caused by several factors. First, the habits of local residents may have an effect. In recent years, the numbers of Toy poodles, Pomeranians, and Golden Retrievers in Xi’an have increased, and the prevalence of dog ear diseases in Beijing has also increased significantly. Second, some hairy breeds, such as Schnauzers and Toy poodles, get regular haircuts. The force of plucking hair from the ear canal destroys the stratum corneum of the ear canal skin, thereby compromising skin integrity and increasing susceptibility to ear diseases.

## Conclusion

Our study analyzed the prevalence of external otitis, ear hematoma, otitis media, and inner otitis in Northwest China. Our findings are conducive to create a profile of canine ear diseases for the area, thereby expanding the current understanding of the development and epidemiology of canine ear diseases and providing a reference for clinical diagnosis, treatment, and prevention.

## Authors’ Contributions

DZL: Designed the study and revised the manuscript. JPL: Analyzed the data. LYL and JPL: Analyzed the results and wrote the manuscript. FLT: Collected the experimental data. All authors interpreted the data, critically revised the manuscript for important intellectual contents, and approved the final version.
